# Calcutta Homœopathic Charitable Dispensary

**Published:** 1893-01

**Authors:** D. N. Banerjee

**Affiliations:** 43 Chorebagan, Calcutta, India


### CALCUTTA HOMŒOPATHIC CHARITABLE DISPENSARY.

43 Chorebagan,

Calcutta, India, September 28th, 1892.

Editor of The Homœopathic Physician:—It gives me pleasure to inform you that Professor J. P. Sutherland, the American Representative of this Charitable Dispensary, has presented several volumes of very valuable works to the library of this institution.

Please give this news a little space in your widely-circulated journal with your favorable remarks to induce others of our colleagues to imitate the noble example of Professor Sutherland.

Yours,

D. N. Banerjee.

